# Dynapenia is highly prevalent in older patients with advanced idiopathic pulmonary fibrosis

**DOI:** 10.1038/s41598-021-97424-y

**Published:** 2021-09-09

**Authors:** Marialuisa Bocchino, Paola Alicante, Ludovica Capitelli, Anna Agnese Stanziola, Lorena Gallotti, Ada Di Gregorio, Gaetano Rea, Alessandro Sanduzzi Zamparelli, Luca Scalfi

**Affiliations:** 1 Respiratory Medicine Section, Department of Clinical Medicine and Surgery, Federico II University of Naples, Via S. Pansini 5, 80131 Naples, Italy; 2 Department of Public Health, Federico II University, Naples, Italy; 3 Department of Radiology, Monaldi Hospital, Azienda Ospedaliera dei Colli, Naples, Italy

**Keywords:** Respiratory tract diseases, Risk factors

## Abstract

Body composition and muscle strength are emerging aspects in idiopathic pulmonary fibrosis (IPF) clinical assessment. We aimed to study the relationships of handgrip strength (HGS) with anthropometric variables, body composition, and disease staging, and to evaluate the prevalence of dynapenia in 102 clinically stable IPF patients (70 M; mean age: 69.4 years). Fat-free mass (FFM), skeletal muscle (SM) were estimated with bioimpedance analysis. HGS was measured with a digital handle dynamometer for both dominant and non-dominant body sides. Dynapenia was identified according to six recognized criteria sets. Mean body mass index (BMI) was 28.2 ± 4.7 kg/m^2^, with a prevalence of overweight (BMI > 25 and < 30 kg/m^2^) and obesity (BMI ≥ 30 kg/m^2^) of 35% and 37%, respectively. FFM and SM were greater in males, whereas percentage body fat was higher in women. HGS was higher and declined with age slightly more rapidly in men, showing a stronger correlation with FFM and SM. Dynapenia prevalence ranged from 20.6 to 56.9%, depending on the criteria used, and was more frequent in older patients and advanced disease. Dynapenia is highly prevalent in IPF. HGS is a promising proxy marker of muscle function to be used in clinical evaluation and follow-up programs.

## Introduction

The evaluation of nutritional status is an increasingly important area in the care process of patients with various chronic diseases. Concerning lung diseases, a consistent body of evidence has shown that in patients with chronic obstructive pulmonary disease (COPD) alterations in body composition and impaired muscle function negatively affect pulmonary function, comorbidities, hospitalization, mortality, etc^1,2^.

Idiopathic pulmonary fibrosis (IPF) is a chronic, progressive, and poor-prognosis interstitial lung disease (ILD) of unknown cause whose incidence has steadily increased, varying from 2.8 to 19 cases per 100 ,000 people/year in Europe and North America, respectively^3,4^. Disease behavior is highly variable, with associated comorbidities having a significant prognostic impact^5^. Although IPF remains refractory to treatment, the current availability of anti-fibrotic drugs, i.e., nintedanib and pirfenidone, has contributed to a certain extent to increase life expectancy and improve quality of life by reducing lung function decline over time, as well as the rate of hospitalization and that of acute exacerbations^6^.

According to a recent expert consensus, only a few papers have provided information on the nutritional status of patients with IPF^7^. Of note, body weight loss was found to be an independent predictive factor of reduced survival^7,8^ with a low body mass index (BMI) in the 36 months before the diagnosis associated with increased mortality^9^. On the other hand, a previous large retrospective study found better survival in patients with BMI > 30 kg/m^2^ compared to those with a lower BMI^10^. Looking at body composition, a recent study estimated that about one-third of patients with IPF were malnourished, exhibiting a low fat-free mass (FFM)^11^. Likewise, a high FFM was predictive of better survival^12^, while a worse prognosis was associated with a reduced cross-sectional area of the erector-spinal muscle^13^.

As far as muscle function is concerned, dynapenia (low muscle strength), which is actually a relevant diagnosis in the clinical setting, is defined as the loss of muscle strength associated with aging (or even nutrition-related diseases) not caused by neurological or muscular disorders^14^. In this regard, it is worth recalling that low handgrip strength (HGS), a proxy marker of muscle strength and a major component of sarcopenia^15^, has been related in the elderly to all-cause and disease-specific mortality, future function, bone mineral density, fractures, cognition and depression, and problems associated with hospitalization^16^.

While muscle strength is frequently considered for prognosis purposes in COPD^17^, only a few studies have evaluated HGS in IPF or fibrotic ILD patients^18–20^, with no information on dynapenia, i.e., the age- and/or disease-associated marked loss of muscle strength not caused by neurologic or muscular abnormalities^14^. Kozu et al.^20^ have shown that there was a highly significant inverse correlation between HGS and the degree of dyspnea, while Guler et al.^18^ reported that in fibrotic interstitial lung disease muscle strength was inversely related to age and directly to weight. Very recently, low HGS has been observed on a preliminary basis (in comparison to predicted values) in a small group of patients with IPF^21^. The reduction of the skeletal muscle mass, evaluated by computed tomography (CT), has been associated to HGS, physical performance, dyspnea and survival in IPF patients^22–24^. Finally, reduced physical activity was related to disease severity^17^ and worse prognosis as well^25,26^.

Based on this background, since 2019 we included the assessment of nutritional status, body composition, and muscle strength in the routine clinical evaluation of patients with IPF. Our cross-sectional study was carried out in such patients to analyze in a real-life setting: (a) the variability of HGS and its determinants; (b) the prevalence of dynapenia using different diagnostic criteria, and (c) the relationship of HGS and dynapenia with anthropometric variables, body composition, lung function, disease severity, and anti-fibrotic therapies.

## Results

The main demographic and clinical characteristics of the study population are reported in Table 1. There were no differences between male and female patients in terms of age. Males were heavier (+ 16.7%) and taller (+ 9.7%), while the mean BMI was slightly higher in females (+ 3.2%). Prevalence of underweight patients (BMI < 21 kg/m^2^) was 7.8%, while overweight (BMI > 25 and < 30 kg/m^2^) and obese patients (BMI ≥ 30 kg/m^2^) were 35.3% and 37.3% of the whole study group, respectively. The 70% and 5% of patients were former or current smokers, respectively, with a median value of 25 packs/year smoked (IR 10–48). Systemic arterial hypertension (54%), gastro-esophageal reflux (27%), and type II diabetes (22%) were the most prevalent comorbidities in the group as a whole with no gender differences. Ischemic cardiovascular disease was more prevalent in males (23 vs. 6%, *p* < 0.05) and thyroid disease in females (21 vs. 3%, *p* < 0.01), whereas pulmonary hypertension (14% of cases) was only slightly more frequent in males (16 vs. 9%; *p* = NS). The median disease duration was 14 months (IR 6–32), with most patients under anti-fibrotic treatment with either pirfenidone (n = 44) or nintedanib (n = 49). As shown in Table 1, most patients were in GAP stages II and III (mild to moderate disease) and in TORVAN stage III, in both cases with no significant gender differences.

**Table 1:** Demographic and clinical characteristics of the 102 patients with idiopathic pulmonary fibrosis (IPF) participating in the study.

	Patients with IPF		
	Total (n = 102)	Males (n = 69)	Females (n = 33)
Age (years)	69.4 ± 7.8	69.5 ± 8.8	69.1 ± 5.4
Stature (cm)*	162.0 ± 9.7	166.6 ± 6.7	151.9 ± 7.3
Weight (kg)*	73.9 ± 13.6	77.4 ± 12.9	66.3 ± 12.2
Body mass index (kg/m^2^)	28.2 ± 4.7	27.9 ± 4.3	28.8 ± 5.4
**Weight status, number of patients and (%)**			
Underweight (BMI < 21 kg/m^2^)	8 (8)	5 (7)	3 (9)
Normal weight (BMI > 21 and < 25 kg/m^2^)	20 (20)	13 (20)	7 (20)
Overweight (BMI > 25 and < 30 kg/m^2^)	36 (35)	27 (39)	9 (27)
Obese (BMI ≥ 30 kg/m^2^)	38 (37)	24 (34)	14 (44)
Smoking status (number of patients who were current/former/no smokers, in number or (%))	5/71/26 (5/69/26)	1/59/9 (1/86/13)	4/12/17 (12/36/51)
Disease duration (months)	14.0 (6.3–31.8)	15.5 (9.0–32.5)	10.5 (5.0–29.0)
Anti-fibrotic therapy (number of patients)	93	66	27
Pirfenidone	43	28	15
Nintedanib	49	35	14
GAP stages	47/44/11	25/35/10	22/9/1
Number of patients and (% in stage I/II/III)	(46/43/11)	(36/50/14)	(69/28/3)
TORVAN stages	26/20/41/15	20/13/26/11	6/7/15/4
Number of patients and (% in stage I/II/III/IV)	(25/20/40/15)	(29/19/37/16)	(19/22/47/13)

Data are expressed as mean ± SD, median value (and interquartile range), or frequency, where appropriate.

GAP = gender-age-physiology.

*p < 0.05 between genders.

Lung function parameters are reported for all patients and by gender in Table 2. A mild to moderate restrictive ventilatory pattern with a similar single-breath diffusing lung capacity of the carbon monoxide (DLCO_sb_) deficit was detected with no clinically significant differences between males and females. The six-minute walk distance was available in 92 patients with an overall median distance walked of 363 m (IR 233–528). Male patients (63) walked more meters than females (29), with median values of 424 m (IR 264–528) for men versus 330 m (IR 189–461) for women.

**Table 2:** Lung function in the 102 patients with idiopathic pulmonary fibrosis (IPF) participating in the study.

	Patients with IPF		
	Total (n = 102)	Males (n = 69)	Females (n = 33)
Partial arterial oxygen pressure (mmHg)^a^	71.2 ± 13.4	70.5 ± 13.6	72.7 ± 13.2
Arterial oxygen saturation (%)^a^	94.4 ± 3.7	94.4 ± 3.7	94.7 ± 3.6
Forced vital capacity (% pred)*	75.0 ± 23.7	72.5 ± 22.0	80.4 ± 26.8
Total lung capacity (% pred)*	64.5 ± 17.0	65.9 ± 16.3	60.9 ± 18.3
Residual volume (% pred)*	59.0 ± 22.5	60.3 ± 20.8	55.9 ± 26.4
DLCO_sb_ (% pred)	51.5 ± 18.8	53.0 ± 17.8	48.6 ± 20.7

Data are expressed as mean ± SD.

*DLCO*_*sb*_ single breath diffusion lung capacity for carbon monoxide.

*p < 0.05 between genders.

^a^Measured at rest while patients were breathing in ambient air.

### Body composition

Mean values of FFM (+ 39.6%), FFM index (FFMI) (+ 15.8%), skeletal muscle (SM) (+ 64.7%) and SM index (SMI) (+ 38.4%) were all greater in male patients (Table 3), while percentage body fat was higher in females (+ 41.6%). All these variables did not significantly vary by the GAP or TORVAN stage. Significant low values of FFMI and SMI were observed in 8.8% and 6.8% of the sample. Finally, body composition was not related to any lung function parameter and no differences emerged by comparing patients treated with nintedanib to those taking pirfenidone.

**Table 3:** Body composition and handgrip strength of the 102 patients with idiopathic pulmonary fibrosis (IPF) participating in the study.

	Patients with IPF		
	Total (n = 102)	Males (n = 70)	Females (n = 32)
Fat-free mass (kg)*	50.1 ± 9.1	55.0 ± 5.6	39.4 ± 5.1
Fat-free mass index (kg/m^2^)*	19.0 ± 2.2	19.8 ± 1.8	17.1 ± 1.9
Skeletal muscle mass (kg)*	24.6 ± 6.0	28.0 ± 3.3	17.0 ± 2.7
Skeletal muscle mass index (kg/m^2^)*	9.2 ± 1.7	10.1 ± 1.2	7.3 ± 1.0
Fat mass (kg)*	23.8 ± 8.0	22.4 ± 7.7	26.9 ± 7.8
Percentage body fat (%)*	31.8 ± 7.8	28.1 ± 5.7	39.8 ± 5.6
Maximum handgrip strength (kg)*	29.1 ± 9.6	33.5 ± 7.8	19.5 ± 5.0
Dominant handgrip strength (kg)*	28.5 ± 9.7	32.8 ± 8.2	19.1 ± 4.9
Non-dominant handgrip strength (kg)*	26.8 ± 9.3	30.8 ± 7.9	17.9 ± 4.9

Data are expressed as mean ± SD.

*p < 0.05 between genders.

### Handgrip strength

Table 3 shows that mean values of maximum-HGS (+ 71.8%), dominant (D)-HGS (+ 71.7%), and non-dominant (ND)-HGS (+ 72.1%) were greater in male compared to female patients with IPF (*p* < 0.001). In both genders, D side values were found to be higher compared to those of the ND side. Maximum-HGS declined with age slightly more rapidly in men (− 0.50 kg/year) than women (− 0.41 kg/year), being 30.2 ± 9.8 kg versus 26.4 ± 8.8 kg in patients aged < 75 years and ≥ 75 years, respectively (*p* < 0.001). The differences between genders and age groups persisted even after controlling weight, BMI, or body composition (data not shown).

The associations of HGS with anthropometric variables and body composition are summarized in Table 4. Maximum-HGS, as well as D-HGS and ND-HGS, directly correlated with weight and BMI and more strongly with FFM and SM. As far as pulmonary function is concerned, maximum-HGS correlated with total lung capacity (r = 0.355, *p* < 0.001), but not with the forced vital capacity (FVC), the DLCO_sb_ and the 6 min walk test (6MWT) distance. Also, neither the dyspnea nor the muscular fatigue, estimated with the Borg scale at the beginning and at the end of the 6MWT, correlated with the HGS (data not shown).

**Table 4:** Partial correlation (adjusted for gender) of handgrip strength with general anthropometric variables and body composition in the 102 patients with idiopathic pulmonary fibrosis (IPF) participating in the study.

	Age	Stature	Weight	Body mass index	Fat-free mass	Fat-free mass index	Skeletal muscle mass	Skeletal muscle mass index	Fat mass	Percentage body fat
**Maximum handgrip strength**										
r	− 0.540	0.317	0.439	0.278	0.465	0.224	0.466	0.235	0.389	0.313
*p*	< *0.001*	< *0.001*	< *0.001*	*0.005*	< *0.001*	*0.025*	< *0.001*	*0.018*	< *0.001*	< *0.001*
**Dominant handgrip strength**										
r	− 0.526	0.322	0.425	0.259	0.455	0.207	0.458	0.222	0.373	0.298
*p*	< *0.001*	*0.001*	< *0.001*	*0.009*	< *0.001*	*0.038*	< *0.001*	*0.026*	< *0.001*	*0.002*
**Non-dominant handgrip strength**										
r	− 0.568	0.308	0.409	0.251	0.421	0.179	0.411	0.180	0.371	0.307
*p*	< *0.001*	*0.002*	< *0.001*	*0.011*	< *0.001*	*0.073*	< *0.001*	*0.072*	< *0.001*	*0.002*

Italics values indicate statistically signidficant results

Multiple regression analysis showed that combining gender (beta = 0.588), age (beta = 0.343), and weight (beta = 0.269) accounted for 67.0% of the variance in maximum-HGS (SEE = 5.53 kg; *p* < 0.001) whereas stature and BMI were not recognized as significant predictors. With respect to body composition, SM (beta = 0.462) emerged as the most important predictor along with gender (beta = 0.293) and age (beta = − 0.318), with adjusted R = 0.660 (SEE = 5.61 kg; *p* < 0.001).

When compared to the other patients with IPF, maximum-HGS, expressed as mean ± standard error (SE), was lower in underweight patients (24.8 ± 3.0 kg vs. 29.5 ± 1.0 kg, *p* < 0.05) and also in those with low FFMI (23.5 ± 3.0 kg vs. 29.7 ± 1.0 kg, *p* < 0.05). Furthermore, after adjusting for gender, maximum-HGS (mean ± SE) was higher in patients in the GAP I stage compared to the other ones (30.8 ± 1.1 vs. 27.9 ± 1.0 kg, *p* < 0.05), and the same was true when TORVAN I was compared to TORVAN II-III stages (32.3 ± 1.4 vs. 28.2 ± 0.8 kg, *p* < 0.05); these differences disappeared when data were adjused also for age. On the contrary, no differences came out by comparing patients treated with nintedanib to those taking pirfenidone.

### Dynapenia

**Figure 1:**
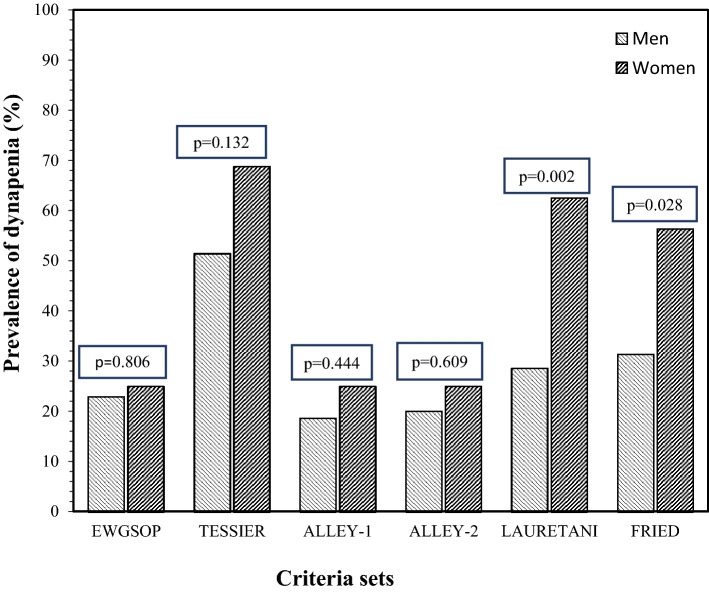
Prevalence of dynapenia (according to different criteria sets) by gender in the 102 patients with idiopathic pulmonary fibrosis (IPF) participating in the study.

Six different criteria sets were used for identifying the dynapenic patients. The prevalence of dynapenia was higher using the TESSIER (56.9%), LAURETANI (39.2%), or FRIED (39.2%) criteria, compared to 23.5% with the EWGSOP-2, 20.6% with the ALLEY-1, and 21.6% with the ALLEY-2 criteria (Fig. 1). The percentage of dynapenic patients did not differ between genders with the EWGSOP-2 (22.9% vs. 25.0%), TESSIER (51.4% vs. 68.8%), ALLEY-1 (18.6% vs. 25.0%) or ALLEY-2 criteria (20.0% vs. 25.0%), but was higher in females than males using the LAURETANI (28.6% vs. 62.5%, *p* < 0.05) or FRIED (31.4% vs. 56.3%, *p* < 0.05) criteria.

**Figure 2:**
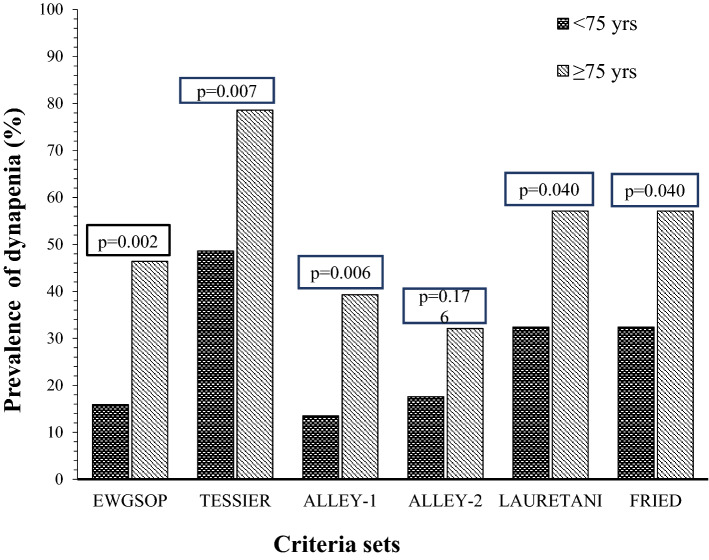
Prevalence of dynapenia (according to different criteria sets) by age groups in the 102 patients with idiopathic pulmonary fibrosis (IPF) participating in the study.

A higher prevalence of dynapenia was observed in the patients with low BMI only using the TESSIER criteria. Those with low FFMI only used the TESSIER or LAURETANI criteria (data not shown). On the contrary, in all cases (except ALLEY-2) dynapenia was much more prevalent in IPF patients aged ≥ 75 years (Fig. 2), for instance, 46.4 versus 15.9% with the EWGSOP-2 criteria and 78.6 versus 48.6% with the TESSIER criteria.

The point prevalence estimate of dynapenia was greater (*p* < 0.05) in GAP stages II-III than GAP stage I with the EWGSOP-2 (32.7 vs. 13.0%), TESSIER (63.6 vs. 47.8%) and ALLEY-1 (27.3 vs. 13.0%) criteria, and in TORVAN stages II-III than TORVAN stage I with the EWGSOP-2 (27.6 vs. 12,0%), LAURETANI (43.4 vs. 24.0%), FRIED (43.4 vs. 24.0%), ALLEY-1 (26.3 vs. 4.0%) and ALLEY-2 (26.3 vs. 4.0%) criteria. Finally, no differences emerged in the prevalence of dynapenia by comparing patients treated with nintedanib to those taking pirfenidone, regardless of the small percentage of cases with mild GI side-effects (14%). Also, no patients reported > 5% weight loss in the three months preceding the study visit.

## Discussion

To the best of our knowledge this is the first report addressing from a clinical point of view the prevalence distribution of dynapenia (i.e., low muscle strength) in IPF patients. It systematically evaluated HGS in this target population, showing lower values in underweight patients or with low FFM. Dynapenia was highly prevalent, even more in patients aged > 75 years. However, the estimated proportion varied depending on the criteria set used.

IPF is a chronic, progressive interstitial lung disease of unknown etiology^3,18^, characterized by a gradual decline of respiratory function up to death. Management and therapy of patients are still complex and not fully defined^27,28^. Disease progression may widely vary among patients: some rapidly deteriorate soon after diagnosis, others maintain a stable condition for years, and still, others show rapid decline following a period of stability^29^.

Alterations of nutritional status and/or body composition, which may negatively affect skeletal muscles, have seldom been described among the systemic manifestations of IPF^30^. To date, BMI has been evaluated in IPF patients with preliminary and sometimes contradictory results^7^; the findings of this study are consistent with previous data from European countries and the United States indicating that the mean value for BMI in patients with IPF was > 25 kg/m^2^^7^. In effect, most of the patients studied were overweight or obese (no one with a BMI > 40 kg/m^2^), while the underweight ones were only 7.8% of the whole study sample. On the contrary, the estimated prevalence of low FFMI, which was around 8%, was less than reported in a recent paper by Jouneau et al*.*^11^; those authors did not give information about the bioimpedance analysis (BIA) equation used to predict FFM, whereas in the present study a well-validated equation for COPD patients was chosen^31^.

As partly surprising and additional information, there was no relationship between FFM and SM with clinical staging systems such as GAP or TORVAN.

Although it is well known that muscle function is related to morbidity, mortality, hospitalization, quality of life, etc. in the elderly and various diseases, i.e., COPD^16,17^, only a few studies have evaluated HGS in IPF patients^18–20^, showing significant correlations of HGS with age (inverse) and weight (direct)^18^. Besides, mean HGS value was found to be lower in more severe patients^19,20^. Very recently, low HGS has been observed on a preliminary basis (in comparison to predicted values) in a small group of patients with IPF^21^. Of note, HGS values largely varied between studies, while the association with body composition has never been taken into consideration, nor dynapenia has been diagnosed. Unfortunately, we do not have direct information of dynapenia in our local population. Indeed, it is interesting to compare our results on HGS with those given in the very relevant paper by Dodds et al*.*^32^ regarding normative values for grip strength in the general population. Twenty-eight per cent of male patients and 25% of female patients with IPF had HGS below the 10th percentiles (for age and gender) reported by Dodds et al*.* and 53% and 63% below the 25th percentiles, thus strongly suggesting low muscle strength. Interestingly, we have derived percentiles for HGS in our local population for adults aged 20–60 years that are very similar to the Dodds ones. In addition, the prevalence of dynapenia in IPF patients is similar to that we have observed in COPD (unpublished results), a chronic lung disease characterized by well-known impairments in the musculoskeletal system.

The findings of our study make it possible to assess the relationships of HGS with various potential predictors. In line with that has been observed in the general population^32^ and COPD patients^33^ HGS showed an inverse correlation with age, as already reported^18^, a direct correlation with weight, FM, and (slightly stronger) with FFM and SM. These observations were confirmed by multiple regression analysis. Models including gender, age explained around 70% of the total variance, and weight or (as an alternative) gender, age and FFM; in other words, no increase in the prediction power was observed substituting FFM for weight. It is also significant to point out that HGS was lower in underweight IPF patients or those with low FFM or low SM. We have also appraised whether muscle strength could be affected by disease severity; quite surprisingly, in line with findings on body composition (see above), we found that HGS did not vary depending on GAP or TORVAN stages, with this finding further confirmed by the weak association found between HGS and lung function.

Dynapenia is defined as the loss of muscle strength associated with aging (or even nutrition-related diseases) not caused by neurological or muscular disorders^14^; the reduction of muscle strength is not necessarily linked to or caused by a decrease in skeletal muscle mass^34^. From a clinical point of view, dynapenia (usually recognized as low HGS values) is associated with reduced ability to perform daily living activities, increased morbidity and mortality, and worse quality of life^15,16^. According to the recent EWGSOP-2 consensus^15^, dynapenia is the first criterion to consider for the diagnosis of sarcopenia. Considering chronic lung diseases, there are few reports on dynapenia in COPD patients^35,36^ and none in patients with IPF. Facing this background, one of the main objectives of the present study was to evaluate the prevalence of dynapenia according to various cutoffs proposed by the literature; in all cases, a subject/patient is dynapenic if his/her HGS falls below a defined threshold value. The cutoffs have been derived in different ways: for example, the recent 2019 EWGSOP consensus^20^ set threshold values based on data published by Dodds et al.^32^ as mean HGS of young adults minus 2.5 standard deviations (SD), while Lauretani et al.^37^ referred to the ability of HGS to discriminate elderly individuals with reduced mobility. Consequently, although there is a certain similarity, the selected cutoffs vary from 26 to 33.1 kg for men and from 16 to 21 kg for women (see “Methods” section).

At first glance, mean HGS values of patients with IPF appear to be relatively low; in effect, the prevalence of dynapenia was high, but indeed varied considerably according to the selected criteria both in male(from 20.0 to 51.4%) and female patients (from 25.0 to 68.8%), with a tendency towards higher values in the latter. There was a clear effect of age as dynapenia was significantly more often detected in patients aged 75 years or more; for instance, the prevalence increased from 15.9 to 46.4% with the EWGSOP-2 criteria and from 48.6 to 78.6% with the TESSIER criteria. These findings are not unexpected since a decline of HGS with aging is a well-known phenomenon^15,38^,while the cutoffs are the same for all subjects. On the other hand, it is worth noting that in most instances, the prevalence of dynapenia did not differ in underweight patients or those with low FFM. Finally, the comparison concerning the severity of disease, dynapenia was more prevalent in the more advanced GAP or TORVAN stages. Overall, as recently stated^21^, and based on what is known for COPD, the impairment of muscle function in patients with IPF is likely to be multifactorial and due to muscle disuse, hypoxaemia, inflammation, oxidative stress, etc. Dynapenia seems to be a relevant clinical problem especially in older patients.

Limitations of our study include the single-center setting along with the cross-sectional and retrospective design. The study population included patients intercepted at different time points of the disease, and most of them were already on anti-fibrotic therapy. Unfortunately, the sudden occurrence of the COVID-19 pandemic prevented any effort of recruitment and follow-up of new cases. Longitudinal studies will help understand the impact of dynapenia and altered body composition on disease presentation and progression/survival, maybe in combination with the quality of life evaluation. An additional limitation of our study is the lack of data on physical activities. Certainly, future efforts will help address this issue as well. Any inter-relation with anti-fibrotic therapies also merits to be further investigated. With respect to this topic, although we found no associations of either body composition, HGS, and dynapenia with the currently used drugs, nintedanib and pirfenidone, to the best of our knowledge, the present study represents the first attempt in exploring this area. Recently, Jouneau et al*.* have shown, by retrospectively evaluating the pooled data of the two Inpulsis trials, that the rate of FVC decline was greater in patients with low baseline BMI and those with > 5% weight loss over 52 weeks. Interestingly, nintedanib reduced lung function decline independently of BMI and had a greater effect in patients with > 5% weight loss^8^. In addition, Suzuki et al. have shown in two retrospective case series that IPF patients under anti-fibrotic therapy had skeletal muscle loss and that sarcopenia was a prognostic factor of reduced survival^39^.

In conclusion, the present study systematically evaluated HGS in IPF, showing lower values in underweight patients or with low FFM. HGS varied, at least in part, depending on weight, body composition, and especially age. The age-related decline in muscle strength persisted even after correction for body composition. From a clinical point of view, dynapenia (i.e., low muscle strength) was found to be highly prevalent, even more in patients aged > 75 years, although the estimated proportion varied depending on the criteria set used. Overall, the point prevalence estimate of dynapenia tended to be higher in GAP stages II–III than GAP stage I and in TORVAN stages II–III than TORVAN stage I. Finally, no differences emerged in HGS or prevalence of dynapenia by comparing patients treated with nintedanib to those taking pirfenidone.

Based on the evidence gathered, the measurement of HGS appears to be a promising proxy index of muscle function of IPF patients. Further studies are needed to better understand how this variable can best be used in the disease's clinical management and identify that part of patients that requires special attention in terms of applied nutrition and motor rehabilitation. Endpoints of particular interest would be, along with survival, also physical activity, quality of life, disease progression and anti-fibrotic therapies.

## Methods

### Study population

The study population was composed of 102 (70 males and 32 females) consecutive patients with clinically stable IPF referring to our outpatient clinic from February 1st, 2019, to March 1st, 2020. They included nine treatment naïve IPF patients and 93 patients already on anti-fibrotic therapy with pirfenidone (n = 44) or nintedanib (n = 49). No patients were previously treated or were taking inhaled or systemic corticosteroids. IPF diagnosis was revised in all patients according to the 2018 official diagnostic criteria^3^. Coexistance of paraseptal/centrolobular emphysema was detected in a small percentage of cases (8%). Exclusion criteria were related to the diagnosis of respiratory diseases other than IPF, acute exacerbation in the four weeks before the study visit, and lung cancer coexistence. Additional exclusion criteria were related to osteo-muscular and neurological disorders or presence of pace-maker/implantable cardioverter defibrillator. Hospitalization in the three months preceding the study visit was reported in no cases. The study was retrospectively conducted in accordance with the amended Declaration of Helsinki and was approved by the local Ethics committee (Federico II University. Registration number: 120/2020). Enrolled patients gave their written informed consent to participate in the study, and all data of interest were anonymously collected into a dedicated database. Spirometry, lung volumes measurement, and determination of the hemoglobin (Hb)-adjusted DLCO_sb_ were performed using a computer-assisted spirometer (Quark PFT 2008 Suite Version Cosmed Ltd, Rome, Italy) according to international standards^40–42^. The 6MWT was performed by trained hospital staff according to guidelines in those patients with a basal peripheral oxygen saturation > 90% in ambient air^43^. The Borg scale was used to assess the level of dyspnea and muscular fatigue at the beginning and at the end of the test. The GAP (stages I, II and III) was recorded as previously described. Accordingly, the TORVAN (stages I, II, III and IV) score, which is a disease complexity index accounting for comorbidities that impact on IPF prognosis, was evaluated as well^44,45^.

### Anthropometry and body composition

Body weight and stature were measured to the nearest 0.1 kg and 0.5 cm with a mechanical column scale and a stadiometer, respectively (SECA 711 and SECA 220, Hamburg, Germany); BMI was then calculated as body weight (in kg) divided by stature squared (in m^2^). Body composition was assessed by BIA. Measurements were carried out with a Human Im-Touch analyzer (© DS Medica S.r.l., Milan, Italy) in standardized conditions (i.e., ambient temperature 23–25 °C, fast > 4 h, empty bladder, supine position for at least 10 min before testing). After cleaning the skin surface, the patients were asked to lie down with their legs and arms slightly abducted to avoid any contact between the limbs and the trunk. A standard tetra-polar technique was used: measuring electrodes were placed on wrist and ankle dorsal surface, while injecting electrodes were on the dorsal surface of the hand and the foot, respectively. Impedance (Z) was measured for both the D and ND body side with an electrical current of 800 mA. Concerning BIA-based estimates of body composition, FFM and SM were determined using BIA equations proposed by Rutten et al.^31^ for patients with COPD and by Jenssen et al.^46^, respectively. FFMI was calculated as FFM/stature^2^ and SMI as SM/stature^2^, while fat mass (FM) was obtained by subtracting FFM from the weight.

### Muscle strength

HGS was measured by the same operator following standard procedures using a Dynex dynamometer (MD systems Inc. Ohio USA) to assess the isometric strength of the D and ND arm. Patients were instructed to stand upright with their shoulder adducted and neutrally rotated, elbow fully extended, and forearm and wrist neutrally positioned during the study. A pre-test was done, allowing the patient to become familiar with the instrument. Three measurements were performed for each hand, one minute apart, alternating between the dominant and non-dominant sides^47,48^. Maximum values were derived for each arm (D-HGS and ND-HGS), and maximum HGS was finally derived as the highest value of six attempts.

### Dynapenia

Dynapenia was defined according to six different criteria sets, five of which derived from consensus documents on the diagnosis of sarcopenia: the FRIED^49^ and LAURETANI^37^ criteria were proposed by the 2010 EWGSOP consensus^50^, the ALLEY-1 and ALLEY-2 criteria^38^ by the FNIH Sarcopenia Project^51^, and the EWGSOP-2 criteria by the corresponding 2019 consensus^15^, based on data by Dodds et al.^32^. In addition, the TESSIER criteria^52^, which were recently established in a large sample of Canadian population, were also selected. Whatever the criteria used, a subject/patient is dynapenic if his/her HGS falls below a defined threshold value. The cutoff values for various criteria sets were as follows.

**Table:** 

Criteria set	MEN	WOMEN
EWGSOP-2 (2019)	< 27.0 kg	< 16.0 kg
TESSIER (2019)	< 33.1 kg	< 20.4 kg
ALLEY-1 (2014)	< 26 kg	< 16 kg
ALLEY-2 (2014)	< 1.0 (calculated as HGS/BMI)	< 0.56 (calculated as HGS /BMI)
LAURETANI (2003)	< 30 kg	< 20 kg
FRIED (2001)	BMI ≤ 24 kg/m^2^: HGS < 29 kg	BMI ≤ 23 kg/m^2^:HGS < 17 kg
	BMI > 24 to 26 kg/m^2^:HGS < 30 kg	BMI > 23 to 26 kg/m^2^: HGS < 17.3 kg
	BMI > 26 to 28 kg/m^2^:HGS < 30 kg	BMI > 26 to 29 kg/m^2^: HGS < 18 kg
	BMI > 28 kg/m^2^: HGS < 32 kg	BMI > 29 kg/m^2^: HGS < 21 kg

### Statistical analysis

Results are expressed as mean ± SD or SE, median value (and interquartile range = IR), and frequency, where appropriate. Statistical significance was pre-determined as *p* < 0.05. All statistical analyses were performed using the Statistical Package for Social Sciences (SPSS Inc, Chicago, IL, USA) version 24. ANOVA with the post-hoc Tukey test and the general linear model (GLM) were used to compare groups and assess the effects of factors on a single dependent variable (in the case, even after adjusting for covariates). Partial correlation and multiple regression analysis were utilized to identify predictors of a given dependent variable.

### Ethics approval and consent for publication

The Ethics committee approved the study of the Federico II University of Naples, Italy (Registration number: 120/2020; 22/05/2020). All patients signed informed consent.

## Data Availability

Data are available upon reasonable request.

## Abbreviations

BIA: Bioimpedance analysis
BMI: Body mass index
COPD: Chronic obstructive pulmonary disease
CT: Computed tomography
D: Dominant side
D-HGS: Handgrip strength dominant side
DLCO_sb_: Diffusion lung capacity for carbon monoxide_single breath_
FFM: Fat-free mass
FFMI: Fat-free mass index
FM: Fat mass
FVC: Forced vital capacity
GLM: General linear model
HGS: Handgrip strength
ILD: Interstitial lung disease
IPF: Idiopathic pulmonary fibrosis
IR: Interquartile range
Maximum HGS: Greatest value for HGS considering both arms
6MWT: 6 min Walk test
ND: Non-dominant side
ND-HGS: Handgrip strength non-dominant side
SD: Standard deviation
SE: Standard error
SEE: Standard error of estimate
SM: Skeletal muscle
SMI: Skeletal muscle index
Z: Impedance

## References

[1] Long R, Stracy C, Oliver MC (2018). Nutritional care in chronic obstructive pulmonary disease. Br. J. Community Nurs..

[2] Raad S, Smith C, Allen K (2019). Nutrition status and chronic obstructive pulmonary disease: Can we move beyond the body mass index?. Nutr. Clin. Pract..

[3] Raghu G (2018). Diagnosis of idiopathic pulmonary fibrosis. An official ATS/ERS/JRS/ALAT clinical practice guideline. Am. J. Respir. Crit. Care Med..

[4] Hutchinson J, Fogarty A, Hubbard R, McKeever T (2015). Global incidence and mortality of idiopathic pulmonary fibrosis: A systematic review. Eur. Respir. J..

[5] Kreuter M (2016). Impact of comorbidities on mortality in patients with idiopathic pulmonary fibrosis. PLoS ONE.

[6] Maher TM, Strek ME (2019). Antifibrotic therapy for idiopathic pulmonary fibrosis: Time to treat. Respir. Res..

[7] Faverio P (2020). Nutrition in patients with idiopathic pulmonary fibrosis: Critical issues analysis and future research directions. Nutrients.

[8] Jouneau S (2020). Analysis of body mass index, weight loss and progression of idiopathic pulmonary fibrosis. Respir. Res..

[9] Kim JH, Lee JH, Ryu YJ, Chang JH (2012). Clinical predictors of survival in idiopathic pulmonary fibrosis. Tuberc. Respir. Dis. (Seoul).

[10] Alakhras M, Decker PA, Nadrous HF, Collazo-Clavell M, Ryu JH (2007). Body mass index and mortality in patients with idiopathic pulmonary fibrosis. Chest.

[11] Jouneau S (2019). What are the best indicators to assess malnutrition in idiopathic pulmonary fibrosis patients? A cross-sectional study in a referral center. Nutrition.

[12] Nishiyama O (2017). Fat-free mass index predicts survival in patients with idiopathic pulmonary fibrosis. Respirology.

[13] Suzuki Y (2018). Distinct profile and prognostic impact of body composition changes in idiopathic pulmonary fibrosis and idiopathic pleuroparenchymal fibroelastosis. Sci. Rep..

[14] Clark BC, Manini TM (2012). What is dynapenia?. Nutrition.

[15] Cruz-Jentoft AJ (2019). Sarcopenia: Revised European consensus on definition and diagnosis. Age Ageing.

[16] Bohannon RW, Wang YC, Yen SC, Grogan KA (2019). Handgrip strength: A comparison of values obtained from the NHANES and NIH toolbox studies. Am. J. Occup. Ther..

[17] Massierer D, Alsowayan W, Lima VP, Bourbeau J, Janaudis-Ferreira T (2020). Prognostic value of simple measures of physical function and muscle strength in COPD: A systematic review. Respir. Med..

[18] Guler SA, Hur SA, Lear SA, Camp PG, Ryerson CJ (2019). Body composition, muscle function, and physical performance in fibrotic interstitial lung disease: A prospective cohort study. Respir. Res..

[19] Hanada M (2016). Effect of long-term treatment with corticosteroids on skeletal muscle strength, functional exercise capacity and health status in patients with interstitial lung disease. Respirology.

[20] Kozu R, Jenkins S, Senjyu H (2014). Evaluation of activity limitation in patients with idiopathic pulmonary fibrosis grouped according to Medical Research Council dyspnea grade. Arch. Phys. Med. Rehabil..

[21] Kanjrawi AA, Mathers L, Webster S, Corte TJ, Carey S (2021). Nutritional status and quality of life in interstitial lung disease: A prospective cohort study. BMC Pulm. Med..

[22] Ebihara K (2021). Appendicular skeletal muscle mass correlates with patient-reported outcomes and physical performance in patients with idiopathic pulmonary fibrosis. Tohoku J. Exp. Med..

[23] Moon SW (2019). Thoracic skeletal muscle quantification: Low muscle mass is related with worse prognosis in idiopathic pulmonary fibrosis patients. Respir. Res..

[24] Nakano A (2020). Early decrease in erector spinae muscle area and future risk of mortality in idiopathic pulmonary fibrosis. Sci. Rep..

[25] Nishiyama O (2018). Physical activity in daily life in patients with idiopathic pulmonary fibrosis. Respir. Investig..

[26] Nolan CM (2018). Phenotypic characteristics associated with slow gait speed in idiopathic pulmonary fibrosis. Respirology.

[27] Sgalla G (2018). Idiopathic pulmonary fibrosis: Pathogenesis and management. Respir. Res..

[28] Millan-Billi P, Serra C, Alonso Leon A, Castillo D (2018). Comorbidities, complications and non-pharmacologic treatment in idiopathic pulmonary fibrosis. Med. Sci. (Basel).

[29] Nakatsuka Y (2018). The clinical significance of body weight loss in idiopathic pulmonary fibrosis patients. Respiration.

[30] Gea J, Sancho-Munoz A, Chalela R (2018). Nutritional status and muscle dysfunction in chronic respiratory diseases: Stable phase versus acute exacerbations. J. Thorac. Dis..

[31] Rutten EP, Spruit MA, Wouters EF (2010). Critical view on diagnosing muscle wasting by single-frequency bio-electrical impedance in COPD. Respir. Med..

[32] Dodds RM (2014). Grip strength across the life course: Normative data from twelve British studies. PLoS ONE.

[33] de Blasio F (2017). Raw BIA variables are predictors of muscle strength in patients with chronic obstructive pulmonary disease. Eur. J. Clin. Nutr..

[34] Sampaio RAC, Sewo Sampaio PY, Uchida MC, Arai H (2020). Management of dynapenia, sarcopenia, and frailty: The role of physical exercise. J. Aging Res..

[35] Martinez CH (2017). Handgrip strength in chronic obstructive pulmonary disease. Associations with acute exacerbations and body composition. Ann. Am. Thorac. Soc..

[36] Strandkvist V (2020). Hand grip strength is associated with fatigue among men with COPD: Epidemiological data from northern Sweden. Physiother. Theory Pract..

[37] Lauretani F (2003). Age-associated changes in skeletal muscles and their effect on mobility: An operational diagnosis of sarcopenia. J. Appl. Physiol..

[38] Alley DE (2014). Grip strength cutpoints for the identification of clinically relevant weakness. J. Gerontol. A Biol. Sci. Med. Sci..

[39] Suzuki Y (2021). Cause of mortality and sarcopenia in patients with idiopathic pulmonary fibrosis receiving antifibrotic therapy. Respirology.

[40] Miller MR (2005). Standardisation of spirometry. Eur. Respir. J..

[41] Wanger J (2005). Standardisation of the measurement of lung volumes. Eur. Respir. J..

[42] Macintyre N (2005). Standardisation of the single-breath determination of carbon monoxide uptake in the lung. Eur. Respir. J..

[43] Laboratories, A. T. S. C. o. P. S. f. C. P. F (2002). ATS statement: Guidelines for the six-minute walk test. Am. J. Respir. Crit. Care Med..

[44] Ley B (2012). A multidimensional index and staging system for idiopathic pulmonary fibrosis. Ann. Intern. Med..

[45] Torrisi SE (2019). The added value of comorbidities in predicting survival in idiopathic pulmonary fibrosis: A multicentre observational study. Eur. Respir. J..

[46] Janssen I, Baumgartner RN, Ross R, Rosenberg IH, Roubenoff R (2004). Skeletal muscle cutpoints associated with elevated physical disability risk in older men and women. Am. J. Epidemiol..

[47] Gerodimos V, Karatrantou K, Psychou D, Vasilopoulou T, Zafeiridis A (2017). Static and dynamic handgrip strength endurance: Test–retest reproducibility. J. Hand Surg. Am..

[48] Roberts HC (2011). A review of the measurement of grip strength in clinical and epidemiological studies: Towards a standardised approach. Age Ageing.

[49] Fried LP (2001). Frailty in older adults: Evidence for a phenotype. J. Gerontol. A Biol. Sci. Med. Sci..

[50] Cruz-Jentoft AJ (2010). Sarcopenia: European consensus on definition and diagnosis: Report of the European Working Group on sarcopenia in older people. Age Ageing.

[51] Studenski SA (2014). The FNIH sarcopenia project: Rationale, study description, conference recommendations, and final estimates. J. Gerontol. A Biol. Sci. Med. Sci..

[52] Tessier AJ, Wing SS, Rahme E, Morais JA, Chevalier S (2019). Physical function-derived cut-points for the diagnosis of sarcopenia and dynapenia from the Canadian longitudinal study on aging. J. Cachexia Sarcopenia Muscle.

